# Cell type-specific changes identified by single-cell transcriptomics in Alzheimer’s disease

**DOI:** 10.1186/s13073-022-01136-5

**Published:** 2022-11-30

**Authors:** Tain Luquez, Pallavi Gaur, Ivy M Kosater, Matti Lam, Dylan I Lee, Jason Mares, Fahad Paryani, Archana Yadav, Vilas Menon

**Affiliations:** Center for Translational & Computational Immunology, Department of Neurology and Taub Institute for Research on Alzheimer’s Disease and the Aging Brain, Columbia University Irving Medical Center, New York, NY USA

## Abstract

The rapid advancement of single-cell transcriptomics in neurology has allowed for profiling of post-mortem human brain tissue across multiple diseases. Over the past 3 years, several studies have examined tissue from donors with and without diagnoses of Alzheimer’s disease, highlighting key changes in cell type composition and molecular signatures associated with pathology and, in some cases, cognitive decline. Although all of these studies have generated single-cell/nucleus RNA-seq or ATAC-seq data from the full array of major cell classes in the brain, they have each focused on changes in specific cell types. Here, we synthesize the main findings from these studies and contextualize them in the overall space of large-scale omics studies of Alzheimer’s disease. Finally, we touch upon new horizons in the field, in particular advancements in high-resolution spatial interrogation of tissue and multi-modal efforts—and how they are likely to further advance mechanistic and target-selection studies on Alzheimer’s disease.

## Background

Alzheimer’s disease (AD) is a progressive neurodegenerative disease with associated brain pathologies, ultimately leading to cognitive decline and dementia [1–3]. The late-onset form of AD (LOAD) is the most common cause of dementia in elderly individuals, and its prevalence is on the rise in many countries with aging populations, resulting in burdens on national health systems. Currently, there are limited therapeutic options for slowing the progression of AD, and these mainly involve cholinesterase inhibitors aimed at slowing down synapse deterioration or the clearance of hallmark pathologies associated with the disease [4]. Whereas pathological features associated with the disease have been known for decades, recent advances in high-throughput, large-scale profiling of brain tissue have allowed for a better understanding of the molecular changes underlying AD [5–10]. The synthesis of information obtained through these techniques is crucial to identifying and prioritizing new candidate targets for additional therapeutic approaches.

Currently, a definitive diagnosis of AD includes ante-mortem cognitive decline leading to dementia, combined with the observation of two major proteinopathies in post-mortem brain tissue [11–15]. These two proteinopathies are plaques, formed by the aggregation of amyloid beta proteins, and tangles, which are composed of hyperphosphorylated tau protein. The importance of these pathological features has been highlighted by studies identifying variants in genes associated with these proteins to be risk factors for dementia [16]. However, the interplay between amyloid, tau, and cognitive decline is complex. These proteinopathies, particularly tangles, follow stereotyped spread through the brain, as evidenced by cross-sectional analysis of post-mortem tissue [17]. This has led to the CERAD, Thal, and Braak staging paradigms, encompassing both quantitative as well as regional examination of these protein aggregates as measures of pathological severity [3, 18–20]. Until recently, however, the cell type-specific effects of this pathological burden have remained underexplored.

**Fig. 1:**
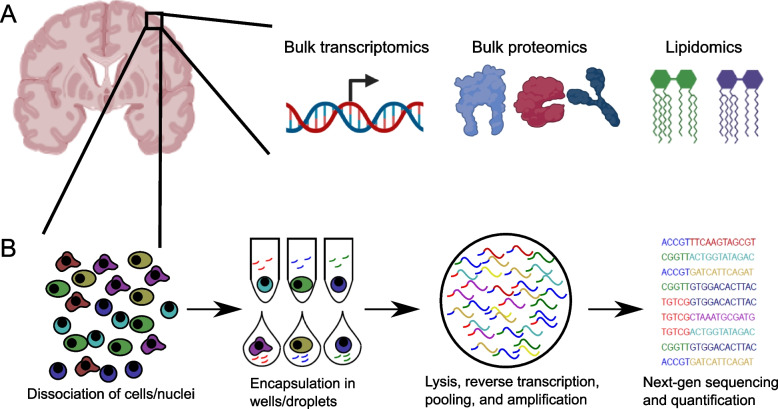
**A** Current bulk profiling approaches that have generated large-scale data from post-mortem human brain tissue in the context of AD. Single-cell approaches have recently been scaled up to allow for a similar type of profiling, but have not reached the donor numbers interrogated using bulk techniques. **B** General workflow for single-cell approaches applied to human brain tissue. After isolation, individual cells or nuclei are encapsulated into wells or droplets, where lysis, reverse transcription, and amplification occurs, usually with some degree of pooling. Barcoded cellular/nucleus cDNA is then sequenced, and reads can be demultiplexed by cell/nucleus barcode and aligned to the transcriptome, ultimately generating expression profiles for each cell/nucleus. Panel **A** was created using the BioRender package

In the past decade, large-scale profiling of bulk post-mortem brain tissue has identified a variety of signatures associated with clinicopathological characteristics. In particular, efforts such as the National Institute of Health’s Accelerating Medicine Partnership-Alzheimer’s Disease (AMP-AD) and the Alzheimer’s Disease Sequencing Project (ADSP) have generated a wealth of bulk profiling data from thousands of brain tissue samples (Fig. 1); this has allowed researchers to identify signals associated with LOAD pathology and LOAD-associated cognitive decline [5–7, 9, 21–23]. Together with large-scale GWAS studies [16, 24], these efforts have implicated glial classes such as microglia, astrocytes, and oligodendrocytes as major players, with dysregulation in their interaction and signatures being strongly associated with the advanced neurodegeneration seen in the disease.

## Experimental and analytic pipeline for single-cell RNA-seq data

Recently, the advent of single-cell/nucleus RNA sequencing (sc/snRNA-seq) has allowed banked and fresh human brain tissue to be profiled at high resolution, leading to deeper characterization of individual cell types and subtypes in the context of AD. Because single-cell studies do not average signals across multiple cell types in a single sample, they allow both the assignment of existing bulk-derived signals to specific cell types, as well as the discovery of novel signals in rarer cell types.

The general procedure for single-cell profiling starts with tissue acquisition (Fig. 1), which then determines whether cell bodies or nuclei are to be isolated. For fresh human brain tissue, protocols have been developed to isolate live microglia and lymphocytes, allowing for scRNA-seq profiling of these cell classes; further protocol development for additional cell classes is underway. For frozen adult human brain tissue, no robust techniques exist yet for single-cell isolation that preserves RNA integrity; as a result, all studies to date on frozen samples have employed snRNA-seq. In contrast to scRNA-seq, snRNA-seq does not profile somatic cytoplasmic transcripts, which may lead to poorer detection of certain transcripts in cell types such as microglia [25]. However, side-by-side studies of the two approaches [26, 27] have shown that the overall distribution of transcripts in the nucleus is sufficient to recapitulate cell type signatures found by scRNA-seq, even with the reduced transcript detection in cell classes such as microglia [28]. It is important to note that additional tissue- and protocol-specific properties might hamper cell recovery and fidelity of the captured transcriptional signature. Thus, specific dissociation protocols must be used to improve yield [29, 30], and artifacts introduced during tissue preparation and cell preparation can be circumvented by using low-temperature non-enzymatic dissociation protocols or inhibiting glial activation [31–33].

After tissue processing, individual cells and nuclei are encapsulated into wells, tubes, or barcoded droplets (Fig. 1), with the latter being used most often for large-scale studies. Reverse transcription then proceeds within each encapsulated volume, followed by amplification of cDNA and next-generation sequencing. Finally, sequencing reads are then aligned to the human genome and transcriptome—modified to include introns in single-nucleus RNA-seq studies to account for a larger fraction of immature transcripts—and quantified, together with barcode-based deconvolution, to yield per-cell expression count values across the transcriptome. The implementation of this protocol at scale on commercialized platforms has revolutionized the study of healthy and disease human brain tissue [34, 35] and is now carried out by many groups studying AD.

**Fig. 2:**
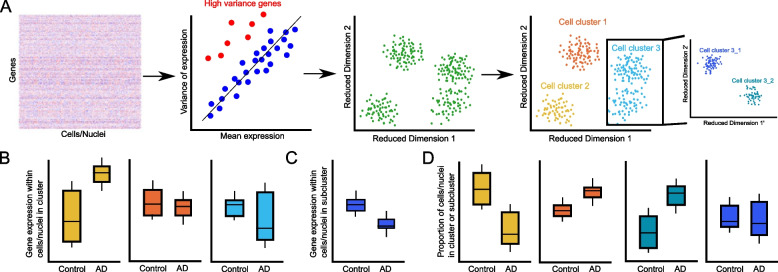
General analytical workflow for large-scale single-cell/nucleus RNA-seq data from human control and AD samples. **A** Starting from the genes × cells/nuclei counts table, most analysis workflows identify high variance genes, then perform dimensionality reduction, and ultimately call clusters in reduced-dimension space. This clustering may be iterative, where larger clusters are then re-analyzed, starting from the first step, to identify subgroups (enlarged inset on the right). It is important to note that often a separate reduced-dimension embedding is used for visualization, as opposed to the embedding used for clustering. **B**–**D** After clusters have been identified, the analysis workflow looks to identify differences in gene expression across conditions in each major cell class (**B**) or subcluster (**C**) or in the relative proportions of each cell class or cluster across conditions (**D**). These differences form the basis of understanding cell type-specific changes associated with the disease

The scale and complexity of sc/snRNA-seq data have required the development of new analytical and computational workflows to identify cell type-specific signatures. After count matrices have been generated, the data analysis workflow first aims to identify putative cell types by clustering individual cells/nuclei based on the similarity of their expression signatures. Currently, a wealth of methods exist for sc/snRNA-seq data clustering, but they generally follow the same overall procedure (Fig. 2): (1) Cells/nuclei are filtered by quality control measures, such as percentage of mitochondrial and/or stress-related genes, total transcript/gene counts, and putative mixed signatures indicative of doublets or contamination; (2) highly variable genes are identified, based on heuristic or distributional measures of gene-specific means and variances; (3) the overall dimensionality of the data is reduced, using methods including principal component analysis, gene clustering, or neural network-based models such as autoencoders; (4) the individual cells are clustered into groups in reduced dimension space using correlation-based clustering methods, network community detection algorithms, or other standard clustering approaches; (5) clusters of cells are characterized to identify marker genes, which can then be used to annotate them based on existing literature, reference atlases, and databases. Often, this entire process is iterated to obtain finer subclusters within each major class of cells (Fig. 2) [26, 29, 34, 36, 37]; these finer subclusters sometimes have new or under-explored markers, leading to the discovery of novel cell signatures. Once this initial clustering approach has created a general taxonomy of putative cell types in the dataset, the disease-focused analysis of cell types then proceeds (Fig. 2); this downstream analysis comprises both differential gene expression within broad classes and subclusters as well as differential cell cluster proportions associated with disease phenotypes. The overall flavor of these analyses can vary among studies, but have still led to the repeated cell type-specific findings described below.

## Limitations of single-cell/nucleus transcriptomics approaches

Despite their tremendous advantages, it is important also to highlight the limitations of and challenges faced by sc/snRNA-seq studies, so as to avoid over-interpretation of results. Regarding completeness, it is important to note that sc/snRNA-seq are unlikely to profile transcripts distal to the soma, especially in cells with highly ramified processes such as neurons. In addition, snRNA-seq, as described above, also excludes transcripts in the soma that are outside of the nucleus. In theory, these sets of missed transcripts are captured in methods that work on bulk tissue, leading to lower sensitivity in sc/snRNA-seq. In addition, the dissociation process may introduce ex vivo artifacts, particularly in live cells when an enzymatic protocol is used, as mentioned above [31, 32]. Fortunately, none of the studies described below is subject to these artifacts, because they do not use enzymatic dissociation protocols. In terms of signal-to-noise ratios, 3′-based profiling methods, such as the one implemented in the widely used 10x Genomics Chromium platform, have low sensitivity for certain genes [38], including potentially lowly expressed but highly relevant ones such as TREM2 in microglia or synaptic genes in neurons. Finally, 3′-based profiling methods (as opposed to more full-length transcript capture methods like SMART-Seq) do not detect reads robustly along the entire transcript and thus have poor detection of alternative splicing and quantification of isoform-specific expression.

On the analytical side, the wealth of tools can be a double-edged sword, offering methodological approaches that suit the most varied experimental designs (Table 1), but may hamper reproducibility and cross-study rigor [39]. Although packages like Seurat and Scanpy have helped unify analytical workflows across studies, there is still room for improvement. For instance, systematic use of strategies like cross-validation and robustness analysis can assess the stability of clustering solutions used to identify cell types, and using appropriate tests of significance can balance false positives and false negatives [39]. With the rapid increase in the range of studies and the number of individual cells/nuclei profiled, the need for standardization is pressing. Finally, it is important to mention the absence of a widely used reference nomenclature for subclusters of cells within all the major classes (excitatory/inhibitory neurons, astrocytes, oligodendrocytes, microglia, vascular cells, etc.). This makes mapping cluster-specific findings across studies challenging and often requires an integrated re-analysis of multiple data sets to identify which observations are reproducible. Indeed, in the synthesis of findings described below, we are restricted to discussing qualitative mapping between cell subclusters in different studies, focusing on specific marker or differential genes highlighted in the text of the studies.

**Table 1:** Summary of recent sc/snRNA-seq and snATC-seq studies on human brain tissue from individuals with AD, with publicly available data

Study	Donors (***n***)	Region	Cohort(s)	Nuclei (***n***)	Modalities	Cell types highlighted
Mathys, Davila-Valderrain, et al. (2019) [40]	48	DLPFC	ROS, MAP	60,462	snRNA-seq	Neu, Oligo, Astro, Mic
Grubman, Chew, Ouyang, et al. (2019) [41]	12	EC	VBB	13,214	snRNA-seq	Oligo, Astr, Mic, OPC
Zhou et al. (2020) [42]	32	PFC	ROS, MAP, Knight-ADRC, BRI	112,809	snRNA-seq	Neu, Oligo, Astro, Mic, Endo
Otero-Garcia et al. (2022) [43]	8	DLPFC	Multiple cohorts	116,684	scRNA-seq	Neu
Cain, Taga, et al. (2020) [28]	24	DLPFC	ROS, MAP	168,713	snRNA-seq	Neu, Oligo, Astro, Mic, Endo
Lau et al. (2020) [44]	20	PFC	SWDBB	167,776	snRNA-seq	Neu, Oligo, Astro, Mic, Endo
Olah, Menon, et al. (2020) [29]	17	DLPFC, TC	ROS, MAP	16,242	scRNA-seq	Mic
Leng, Li, et al. (2021) [45]	10	SFG, EC	NDBB, BBAS	106,136	snRNA-seq	Neu, Oligo, Astro, Mic
Gerrits et al. (2021) [46]	18	OC, OTC	NBB-IBB	482,472	snRNA-seq	Mic, Astro, Endo, Peri
Morabito, Miyoshi, Michael, et al. (2021) [47]	18	PFC	UCI MIND	61,472	snRNA-seq, snATC-seq	Neu, Oligo, Astro, Mic
Brase et al. (2021) [48]	67	PC	Knight-ADRC, DIAN	294,114	snRNA-seq	Neu, Oligo, Astro, Mic
Yang et al. (2022) [30]	26	SFC, H	Stanford/VA/NIA ACRC	143,793	snRNA-seq	Endo, Peri, Fibro
Sadick et al. (2022) [49]	16	PFC	NYU-ADRC, UCSD-ADRC, BTRC	65,180	snRNA-seq	Oligo, Astro

Brain regions: *DLPFC* dorsolateral prefrontal cortex, *EC* entorhinal cortex, *PFC* prefrontal cortex, *TC* temporal cortex, *SFG* superior frontal gyrus, *OC* occipital cortex, *OTC* occipitotemporal cortex, *PC* parietal cortex, *SFC* superior frontal cortex, *H* hippocampus. Cohorts: *ROS* Religious Orders Study at Rush University, *MAP* Memory and Aging Project at Rush University, *VBB* Victorian Brain Bank at the Florey Institute of Neuroscience and Mental Health, *BRI* Brain Research Institute at Niigata University, *SWDBB* South West Dementia Brain Bank, *NDBB* Neurodegenerative Disease Brain Bank at University of California San Francisco, *BBAS* Brazilian BioBank for Aging Studies at the University of Sao Paulo, *NBB-IBB* NeuroBiobank of the Institute Born-Bunge, *UCI MIND* University of California Irvine Institute for Memory Impairments and Neurological Disorders, *Knight-ADRC* Knight Alzheimer Disease Research Center at Washington University, *DIAN* Dominantly Inherited Alzheimer Network, *Stanford/VA/NIA ACRC* Stanford/VA/NIA Aging Clinical Research Center, *NYU-ADRC* NYU Grossman School of Medicine’s Alzheimer’s Disease Research Center, *UCSD-ADRC* University of California San Diego Shiley-Marcos ADRC, *BTRC* Rhode Island Hospital’s Brain Tissue Resource Center

## All major brain cell types show molecular and compositional changes in AD

**Fig. 3:**
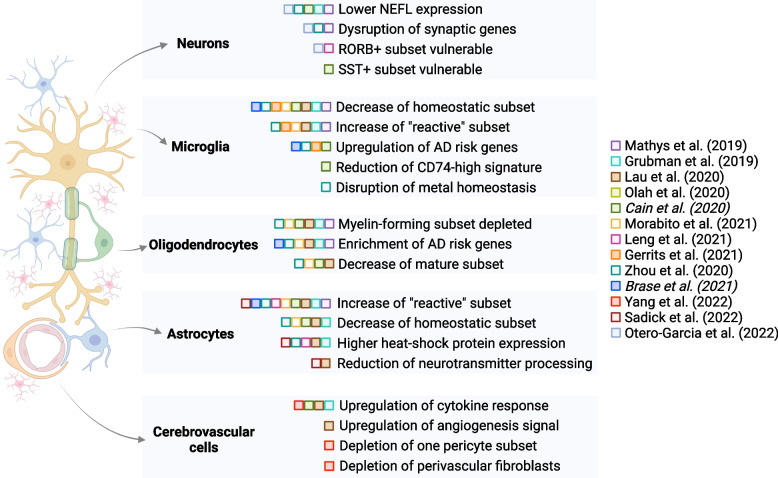
Schematic summary of selected findings from multiple sc/snRNA-seq studies, organized by cell type and publication date (italics indicate the Biorxiv versions of manuscripts). Studies have identified differences in all major cell types in brain tissue from healthy donors versus donors with AD diagnoses. Some of these differences are highlighted only in a subset of studies, suggesting the need for further exploration of reproducibility and consistency of findings. This figure was created using the BioRender package

Here, we survey 13 recent studies that have used sc/snRNA-seq to characterize cell state and composition changes in AD (Table 1). As mentioned above, this task is hampered by the lack of a harmonized nomenclature for cell subclusters, preventing a fully rigorous assessment reproducibility of cell identities. Thus, we summarize concordant compositional (proportion) changes and differential gene expression within cell states across studies, expand on the novel and future technologies that propel them, and offer our perspective on the relevance of single-cell profiling to aid therapeutic development for AD. Notably, this collection of studies implicates changes within all major cell types, emphasizing widespread structural and functional disruption in AD (Fig. 3). Although most studies have focused on the prefrontal cortex, which is affected in the middle-to-late stage of disease progression, we also include studies with findings in early affected limbic and cortical regions when pertinent.

## AD tissue contains higher proportions of microglia with a variety of activated states

Genome-wide association studies and new bulk transcriptomics analyses have identified key AD-specific differences in genes expressed in microglia [16, 24]. Single-cell studies have offered additional insight into microglial heterogeneity in AD, thanks to more sensitive profiling of rare cell types such as microglia, which compose between 5 and 15% of cells in most cortical regions [50]. The first two published snRNA-seq studies on AD both identified a downregulation of homeostatic and cell-cell signaling genes (such as CX3CR1, P2RY12, and P2RY13) in microglia [40, 41]. In parallel, Zhou et al. examined microglia from individuals carrying the TREM2-R47H variant and identified additional upregulated genes in microglia such as SORL1 and CHI3L1, a genetic risk factor and putative biomarker for AD, respectively [42]. Similarly, Morabito et al. reported lower proportions of homeostatic microglia at one end of their microglial pseudotime trajectory, coupled with a concomitant increase of TREM2-independent disease-associated microglia [51]. Finally, Lau et al. showed a lower proportion of microglia with a synaptic pruning signature [44], a prime function of homeostatic microglia.

Concomitant with the decrease in homeostatic signatures, snRNA-seq studies have identified a host of pathways upregulated or dysregulated in human microglia in AD donor tissue. This mirrors recent findings that disease-associated signatures in humans are substantially more heterogeneous than in mouse models on a single genetic background [25, 28, 29, 40, 42, 52]. Zhou et al. found higher proportions of a subtype expressing microglial activation genes such as HLA-DRA, TREM2, and AIF1 in LOAD patient tissue, as well as potential disruption of the metal ion homeostasis pathway [42]. Brase et al. identified that carriers of the MS4A rs1582763 variant, which is associated with reduced AD risk and delayed age of disease onset [53], have higher proportions of a microglial subcluster upregulating pro-inflammatory genes including IL1B, FCGR3A, C3, NPC1, PAG1, and CD40. Interestingly, tissue from these individuals also showed depletion of a different microglial subcluster expressing pro-inflammatory genes (C5, BMPR2, and TPRG1) that overlap with activated-microglial signatures reported previously in neurodegenerative diseases [54] and aging [55]. This highlights the complexity of microglial signatures, as they show differences in the expression of subsets of genes belonging to the same pathway.

Two studies that focused on human microglial through enrichment strategies have further refined subsets of microglia associated with disease trajectory. Gerrits et al. [46], by examining combinations of amyloid and tau proteinopathies, identified pathology-specific states that increase in late stages of the disease. A microglial subset with high expression of MYO1E is specifically associated with amyloid load in the occipital cortex while a different subset with marked expression of CX3CR1 is associated with tau in the occipitotemporal cortex. Finally, Olah et al. used scRNA-seq on microglia isolated fresh autopsy and surgically resected tissue to identify a particular microglial subsignature, marked by high expression of CD74, that was lower in AD donor tissue [29]. An immunohistochemical investigation of post-mortem control and AD tissue confirmed the lower prevalence of this microglial signature in AD. Although this signature is reported in some snRNA-seq studies [28], it remains to be seen whether the association is consistently found in snRNA-seq versus scRNA-seq. Overall, these two studies highlight the importance of both approaches (nuclear and cellular isolation), as well as examining the heterogeneity of pathology distribution in AD, in order to characterize the full complement of microglial signatures associated with disease onset and progression.

In summary, all sn/scRNA-seq studies on AD so far have reported a general reduction of homeostatic signatures in microglia, in combination with dysregulation of a host of pathways. These include “classical” microglial activation signatures, cell-cell signaling, pro- and anti-inflammation, and metal ion homeostasis. These transcriptomic studies, therefore, bolster the GWAS-derived hypothesis of microglial dysregulation playing a major role in AD, while also illustrating the heterogeneity of microglial responses. Although some of these responses could be artifacts related to dissociation, they are not due to enzymatic dissociation protocols, which were not used in any of these studies. Thus, one important next step in synthesizing the diversity of microglial dysregulation in AD is to characterize putative transitions between these microglial states using a combination of continuous modeling approaches and experimental validation using model systems. This next stage of research into microglia in AD will shed further light onto the relationship between AD pathology, progression, and disease-associated microglia signatures.

## Oligodendrocyte signatures in AD are indicative of myelination disruption

As with microglia, GWAS studies have identified AD risk genes implicating dysregulation in oligodendrocytes. Late-onset AD risk genes such as BIN1, PLP1, and CLU are expressed in oligodendrocytes, although the extent to which these specific genes are differentially regulated remained an open question before the advent of snRNA-seq studies. These studies have shown that expression of BIN1 [41, 42], PLP1 [40, 42], and CLU [40–42, 44] are altered in oligodendrocytes in AD, and also highlight a general reduction in myelinating oligodendrocytes in brain tissue from individuals with LOAD. Specifically, myelinating oligodendrocyte signatures are higher in low-pathology individuals and lower in individuals with high pathological burden, when compared to healthy donors [41, 42, 47]. This change in myelination signature with disease progression is also borne out through pseudotime trajectory reconstruction based on gene expression and single-cell ATAC-seq data [47]. Accompanying this ultimate decline in myelinating oligodendrocytes, multiple studies also identified upregulation of stress-response signatures [28, 44, 47]. Importantly, oligodendrocyte dysregulation appears to be a shared feature in both familial and LOAD, as Brase et al. found a higher proportion of an oligodendrocyte subcluster enriched for spliceosome genes highly correlated with the LOAD GWAS hits CLU, MAP1B, and PICALM in carriers of autosomal-dominant AD risk genes APP and PSEN1 [48]. These observations point to a putative shared pathway of dysregulation in specific oligodendrocyte subsets underlying both familial and late-onset AD.

More recently, studies linking oligodendrocyte signatures to genetic variants have implicated potential dysregulation linked to oligodendrocyte-microglial crosstalk in AD. Brase et al. showed carriers of three TREM2 variants associated with reduced microglial activation (R47H, R62H, and H157Y) showed higher proportions of oligodendrocytes marked by upregulation of the myelin biosynthesis repressor TFEB [56]. This TFEB-enriched oligodendrocyte subcluster also showed higher proportions in TREM2-R47H carriers from Zhou et al. [42], supporting the possibility of microglial-oligodendrocyte crosstalk via a TREM2-TFEB pathway. A study focusing on genetics-driven selection of individuals also found an oligodendrocyte subset expressing immune genes (e.g., HLA-A and B2M) in APOE Ɛ2/3 carriers [49]. Although the directionality of this cross-cell type signaling is not established in these studies, the findings point to an oligodendrocyte-microglial axis that is consistently found to be disrupted in AD.

Overall, the two most consistently found oligodendrocyte changes in AD are the reduction in myelin-forming oligodendrocytes and the link between oligodendrocyte signatures and microglial genes. However, it is important to note that quantification and comparison of this cell type across snRNA-seq data sets can be challenging because of variability in the amount of white matter included in the dissection protocol for each study. Whereas dissections are often consistent across samples within the same study, they may vary across studies, thus yielding systematically higher proportions of oligodendrocytes in one study versus another.

**Fig. 4:**
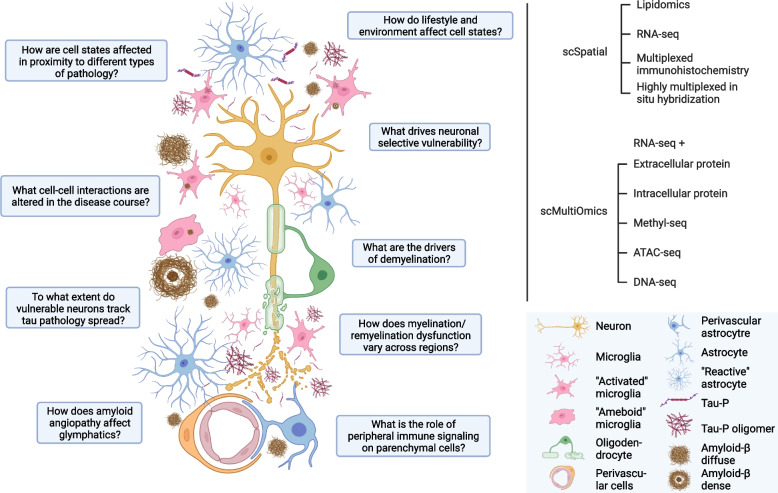
Open questions regarding cell type dysregulation and interactions that have not been extensively addressed by the studies described here. Some of these questions (left) are better served with the integration of data from new modalities and combined approaches (top right). This figure was created using the BioRender package

From a biological perspective, fully recapitulating oligodendrocyte changes along the disease trajectory would benefit from deeper profiling of brain regions affected earlier in the disease progression (like the entorhinal cortex and hippocampus). This approach would also shed light on the potential region specificity of oligodendrocyte subcluster composition, as has been shown for other glial classes such as astrocytes [49, 57]. Given that oligodendrocyte signature changes are found repeatedly in single-cell studies, further examination of this cell class and its interactions with other cell types (Fig. 4) continues to be an active area of research.

## AD astrocyte signatures are altered along multiple spatial, temporal, and functional axes

Astrocytes are central players in coordinating brain homeostasis, from maintaining the blood-brain barrier to preserving neuronal health and excitability. Thus, it is not surprising that multiple studies have identified specific signatures of astrocytes altered in AD. These include higher proportions of astrocyte subclusters with heat-shock protein genes involved in stress response (such CRYAB, HSP1A1, HSBP1, and HSP90AA1) [41, 42, 44, 45, 47, 49], astrogliosis genes (GFAP and VCAN) [28, 40, 44], and regulatory genes implicated in neurodegeneration (NEAT1) [44]. These studies also found concomitantly lower proportions, in individuals with AD, of astrocyte signatures associated with synaptogenesis (NRXN1 and NRXN3) and neurotransmitter balance. These same studies also identified an increase in the proportion of CD44+ astrocytes in AD. CD44+ astrocytes have fibrous-like morphology and are prominent in the white matter [58], but three studies have independently observed an increase in this astrocyte subgroup proportion associated with cognitive decline [28] and AD pathology [28, 45, 49] in the frontal cortex.

The refinement of novel astrocyte signatures has also been facilitated by integrating data from multiple studies. By combining their snRNA-seq with previous studies, Sadick et al. [49] report a putative duality in the astrocytic function that can also be observed in other datasets, where both loss and gain of function signatures coincide in the same cluster. For example, one cluster has higher expression of genes involved in scarring (RGCC) as well as in antioxidant pathways (SOD and MT1G). This functional duality is also seen at the cluster/subcluster level, where many of the astrocytic pathways previously implicated in AD (i.e., metal ion regulation, protein homeostasis, angiogenesis, synaptic maintenance, lipid storage, and fatty acid oxidation) can be ascribed to either subcluster-specific or multi-cluster signatures. Importantly, this integrated analysis using data from multiple studies found shared signatures across all studies after batch correction. This approach highlights the relevance of integrating datasets to assess reproducibility and to power the discovery of disease signatures in novel and established subpopulations.

Finally, the astrocyte signatures in autosomal-dominant AD mirror many aspects of those described in LOAD. Brase et al. [48] showed relatively higher proportions of an astrocyte cluster upregulating VIM, OSMR, and CTSB, like the ones found by Morabito et al. [47], and paralleling plaque-associated astrocytes identified in mouse models as well [59]. In addition, they found that homozygous carriers of MS4A rs1582763 have lower proportions of homeostatic astrocytes and higher proportions of an astrocyte subcluster upregulating the markers of plaque-associated astrocytes (i.e., GFAP, ID3, AQP4, ID1, and GSN) [48]. These findings suggest that different genetic architectures of AD might converge onto overlapping axes of dysregulation in astrocytes.

In sum, these studies suggest that impairment of astrocyte function and transition to a more stress-associated or putatively reactive state are hallmarks of AD. Importantly, the overall heterogeneity of astrocytic signatures goes beyond the earlier A1/A2 neurotoxic/neuroprotective model [60], suggesting that astrocytes in the aging brain are altered along multiple temporal, spatial, and functional axes in the context of disease. This includes changes in classes of astrocytes once thought to be predominantly white-matter associated. An active area of research is discovering the putative associations between these continuous axes of biological variation and pathological load, with the goal of better functional annotation of subclusters (Fig. 4). Given the complexity of the astrocytic transcriptomic landscape, it is likely that further studies will uncover additional signatures that show stage-specific progression in AD.

## Cerebrovascular cells exhibit inflammatory profiles and selective vulnerability in AD

Given the potential dysfunction of the blood-brain barrier in AD, it is expected that vascular cells may show altered signatures in the disease. However, as with microglia, these cells form a small portion of the total population, and subtle signatures may be obscured in bulk profiling data without specific enrichment for these cells. With single-nucleus approaches that isolate sufficient numbers of vascular cells, disease-specific signatures have become easier to assess. Grubman et al. [41] identified the upregulation of cytokine signaling and immune response genes in endothelial cells from the entorhinal cortex, including HLA-E, MEF2C, and NFKBIA. These findings were expanded on by two data sets that included larger numbers of vascular nuclei [28, 44], which identified multiple subsets of endothelia in AD donors that were enriched for genes associated with angiogenesis, such as ERG and VWF, as well as immune response genes such HLA-E and IFI27.

Recently, Yang et al. dramatically enriched vascular cell numbers per donor using a novel method for vessel isolation and nucleus extraction for sequencing (VINE-seq) [30]. This study not only recapitulated the immune signaling gene pathways in endothelial cells reported previously, but also showed that this inflammatory profile is present in venous endothelial cells. Using an endothelial taxonomy based on spatio-functional differences of zonation [61], they mapped this potential immune signaling profile to capillary and arterial endothelial cells specifically in APOE Ɛ4 carriers. Moreover, all three types of endothelial cells (venous, capillary, and arterial) were significantly depleted in AD samples. Among non-endothelial cerebrovascular cell classes, this study found that pericytes expressing genes involved in extracellular matrix reorganization were depleted in AD, whereas pericytes expressing small-molecule transport genes did not show the same extent of depletion. Similarly, adjacent mural smooth muscle cells were also lower in AD and had the greatest number of AD upregulated genes of all perivascular cell types. Finally, perivascular fibroblasts, but not those that are meningeal, were also significantly lower in AD. The selective vulnerability of endothelial, mural, and fibroblast cells in AD may reflect the reported structural breakdown of the blood-brain barrier reported in LOAD [62].

The findings from these snRNA-seq studies suggest two main components of vascular dysregulation in AD—upregulation of an inflammatory/immune response signature and selective loss of fibroblast, endothelial, and mural cells. Importantly, these cell subsets also express similar numbers of genes associated with AD risk loci as microglial and myeloid cells [28, 30, 44]. Finally, given the close connection between the cerebral vasculature and the glymphatic system, an area of ongoing research is the link between perivascular composition and alterations in glymphatic function (Fig. 4). On the therapeutic side, the identification of specific subsets of cerebrovascular cells associated with disease, together with surface markers and signaling pathways identified by snRNA-seq studies, may lead to candidate-based approaches to repair dysregulated blood-brain-barrier function in AD.

## Neuronal subtypes show selective vulnerability to tau and neurodegeneration

The ultimate cellular outcome in AD, which is also most proximately related to circuit dysfunction, is neurodegeneration. From a pathological perspective, the accumulation of intracellular tau is primarily restricted to neurons and follows a stereotyped spread in the brain [3]. This selective appearance of pathology suggests that distinct neuronal types may be differentially vulnerable or resistant in the course of the disease. Indeed, brain-mapping studies have identified neuronal subtypes that are likely to be lineage-specified [36]; thus, alterations in neuronal signatures in disease are likely due to selective vulnerability, rather than interconversion between different neuronal classes.

Cortical neurons can be broadly divided into two groups—glutamatergic and GABAergic. Glutamatergic neurons, which can project locally to other neurons or long-range to distant cortical or subcortical regions, show layer-specific expression of marker genes in the cortex [34]. From a transcriptome perspective, they can be identified by the expression of glutamatergic genes such as SLC17A7 (VGLUT1). Since glutamate is primarily (but not exclusively) a mediator of excitatory signaling in the adult cortex, glutamatergic neurons coincide with functional and structural definitions of “excitatory” or “projection” neurons [34, 36]. By contrast, most GABAergic neuron subclasses have local projections and show weak layer-specific enrichment; they coincide with “inhibitory” neurons (“interneurons”) and are marked by expression of genes in the GABAergic pathway, including GAD1, GAD2, and SLC32A1 (VGAT). Brain-mapping studies have shown that neuronal signatures, especially those of glutamatergic (putatively excitatory) neurons, are brain-region specific and differ even among neighboring cortical regions [63]. Although this region specificity is less pronounced for GABAergic (putatively inhibitory) cortical neurons, it is not entirely absent [26, 34, 63]. Thus, given the regional stereotypy of tau pathology spread in AD, it is likely that these two major classes of neurons are affected in different ways in the course of the disease.

An ongoing area of research in AD is the extent to which different neuronal subgroups are vulnerable or resistant to pathology and disease progression. However, bulk studies pose challenges in identifying changes in subtypes of neurons beyond the two major classes described above. By contrast, snRNA-seq studies have identified vulnerability to pathology in deep layer glutamatergic neurons in specific brain regions. Leng et al. [45] showed that RORB+ glutamatergic neurons are selectively depleted in the entorhinal cortex, but not in the superior frontal gyrus, evidence for layer- and subset-specific early-stage neurodegeneration in AD. Similarly, Otero-Garcia et al. [43] used a novel partial cell body sorting technique to identify that a subset of neurons in layer 2/3, as well as a subset of deeper layer RORB+-positive neurons in the frontal cortex, are enriched among cells with higher abundance of intracellular AT8+ tau in their somata. In addition, these vulnerable sets of neurons share a common upregulation of genes encoding for synaptic and cytoskeletal proteins [43], as has been found in other snRNA-seq studies [40, 44]. This study also identified certain RORB+ neuronal subsets as being resistant to the accumulation of tau pathology, whereas the RORB+/PCP4+ subgroup preferentially accumulates tau; this suggests that not all projection neurons, even within the same cortical layer, are equally vulnerable. Thus, these studies now assign specific molecular markers to layer-specific neuronal loss that has been described through careful immunohistochemistry and histology over two decades ago [64, 65]. Overall, these subtype-specific molecular signatures could form the basis for therapeutic avenues focused on protecting vulnerable neuronal subtypes in layers 2/3 and 4, thus mitigating circuit-related and cognitive impairment in AD.

Single-cell studies have also linked neuronal profiles with NEFL and APOE, two genes heavily studied in AD. Multiple snRNA-seq studies have reported the loss of neurons with high expression of NEFL in AD [28, 42, 43]. This corroborates prior histological studies showing alterations in NEFL distribution in intact tissue in individuals with mild and severe AD [64, 65]. In addition, this finding gels with the observation that higher levels of NEFL in cerebrospinal fluid may serve as a biomarker for LOAD progression. With regard to APOE, a gene with risk variants associated with AD, Brase et al. reported higher proportions of neurons expressing the AD-risk-associated gene APOE in autosomal-dominant AD [48]. However, this detection of APOE above background levels has not been consistently found in LOAD studies, suggesting either technical variability across studies or a difference between LOAD and familial AD.

Whereas projection neuron loss has been noted as a hallmark of neurodegeneration and circuit function dysregulation, less has been known about GABAergic neurons in the cortex in AD. In mouse models of AD, for example, GABAergic neurons can exhibit resistance to the spread of tau, particularly in the entorhinal cortex [66]. The extent to which subtypes of GABAergic neurons show differential vulnerability has remained under-explored. Cain et al. [28] suggest there is a selective loss of SST+ neurons in the frontal cortex, with corresponding resistance in PVALB+ neurons. Since both subgroups of neurons originate from the medial ganglionic eminence, this finding suggests that selective vulnerability in GABAergic neurons does not necessarily affect all cell types from a given lineage. Similarly, Brase et al. found that two GABAergic neuron populations showed significant associations to autosomal-dominant AD and also that these subsets might be affected earlier than glutamatergic neurons in this form of the disease [48]. Overall, the impact of AD pathology on GABAergic neurons is under-explored, and further investigation is needed to establish the temporality of how these neurons are selectively affected during the progression of the disease.

On the neuronal front, single-cell studies have provided an unprecedented resolution to study neuronal selective vulnerability in AD. However, important questions remain regarding how neurons are dysregulated and how their interactions with glia shape their vulnerability or resistance through disease (Fig. 4). It is still unclear whether the subset of RORB+ excitatory neurons in the entorhinal cortex, which are depleted in early stages of AD, correspond with the RORB+ subsets harboring tau tangles in the frontal cortex [43]. The extent to which this signature of vulnerability tracks the stereotypical pattern of tau pathology spread throughout the cortex is also unclear. Finally, the implication of subsets of GABAergic neurons, which are known to be dysregulated in neuropsychiatric disease, raises intriguing questions as to how the disruption of excitation and inhibition in the cortex may contribute to cognitive symptoms in AD. Further investigation into these aspects of selective vulnerability, shared signatures across cortical regions, and circuit-level dysfunction is all likely to yield new classes of therapeutic interventions related specifically to neuronal changes in AD.

## Ongoing developments and new technologies

The advent of new technologies, as well as improvements in single-cell approaches, mark a new horizon in the study of neurodegenerative diseases, including AD. Although sc/snRNA-seq techniques have become routine in profiling human brain tissue, they have important limitations. These include the loss of spatial context, reduced sensitivity for certain genes, and some degree of discrepancy with other modalities such as proteomics. One area of explosive growth has been the development of spatial profiling methods, which eliminate the need for tissue dissociation and thus capture disease-associated changes in the native context [67]. Transcriptome-wide approaches such as the 10x Genomics Visium platform (formerly known as Spatial Transcriptomics) and NanoString’s GeoMx DSP, or highly multiplexed in situ hybridization/immunohistochemical methods such as MERFISH, SeqFISH, Nanostring, 4i, and Codex, all allow for the interrogation of multiple RNA or protein species in intact tissue. This allows analysis of not only cell type-specific alterations, but also changes in the arrangement of cell types with respect to each other and with respect to pathological features, both of which are impossible in single-cell approaches requiring dissociation. In mouse tissue, a multiplexed ISH study identified microglial signatures enriched near amyloid beta plaques [68] whereas a large-scale Spatial Transcriptomics study on ALS identified major cell type spatial changes in the human spinal cord [69]. The significant immune compositional changes local to the accumulation of proteinopathies identified by both of these studies provide new insight into potential immune-targeting therapeutic approaches. Thus, as these methods scale with respect to spatial resolution, throughput, and cost, they may ultimately obviate the need for dissociation-based methods to profile tissue at the single-cell level across the transcriptome.

Another axis of investigation being applied to human brain tissue is simultaneous interrogation of the transcriptome and epigenome (Fig. 4). As shown in Table 1, Morabito et al. generated mRNA and ATAC profiles on separate sets of nuclei from the same samples [47] and highlighted the importance of ATAC-seq in linking genetic risk variants to cell type-specific mRNA profiles, as well as oligodendrocyte epigenetic alterations in disease progression. The results agree with previous studies mapping open chromatin around AD risk loci in a cell type-specific way using snATAC-seq alone [70], inferring causal links between genetic risk factors and cell type dysregulation. Newer approaches now exist to profile mRNA and chromatin accessibility in the same nuclei (as opposed to parallel sets from the same sample); this would allow for more direct linking between risk genes, chromatin state, and cell type profiles, leading to potential new insight into changes during earlier stages of the disease. Similarly, single-cell mRNA+DNA methylation platforms in development would allow for alternative measurement of epigenetic profiles, further linking genetic risk factors and cell type-specific transcriptomes. Finally, it has now become possible to study gene perturbation effects through techniques like CROP-seq [71] or Perturb-seq [72], which allow for highly parallelized profiling of dozens to hundreds of genetic perturbations in vitro. Although the in vitro model does not fully recapitulate aging brain cells, it nonetheless provides a model to test hypotheses about causal mutations or gene perturbations on specific cell types. As these multi-modal platforms and methods improve, they thus hold the promise of identifying genetic and epigenetic alterations upstream of transcriptomic changes and potentially link gene-by-environment interactions to cell type-specific transcriptomic changes (Fig. 4).

Finally, the advent of large-scale proteomic methods applied at the single-cell level will provide valuable insight into disease-associated processes and potential therapeutic avenues missed by transcriptomics-only studies. Bulk proteomics studies [73] suggest that certain signals and modules may be missed by corresponding bulk RNA-seq studies. Currently, single-cell proteomics on peripheral cells can quantify hundreds of proteins in an individual cell [74] or both RNA-seq and dozens of surface proteins through CITE-seq [75]. However, for brain tissue, tissue dissociation remains an important challenge for both of these methods; indeed, this challenge cannot be bypassed easily, given that proteins are substantially more dispersed throughout the entire arbor of a cell as compared to RNA. This limitation is circumvented by highly multiplexed immunohistochemical approaches such as 4i and Codex, which interrogate intact tissue. Although these latter methods are currently limited by antibody selection and the degree of multiplexing possible, they show promise in identifying cell type rearrangements as well as cell type-specific protein expression changes in tissue [76, 77]. Together, these advances in proteomics may usher in a new era of disease-specific target identification and protein-based biomarkers and therapeutic modulation of cell types.

The integration of multiple modalities with sc/snRNA-seq has implications not only for tissue profiling, but also for the clinic. Whereas brain tissue itself is not viable as a biomarker for disease staging and assessment in living patients, the application of profiling methods to tissue can yield new candidate biomarkers likely to be detected in more accessible compartments such as CSF. By identifying key genes dysregulated across disease stages in the cross-sectional studies described here, these new approaches may help refine the set of biomarkers for AD stratification and progression. In parallel, the characterization of vulnerable and resistant cell types, together with upregulated and downregulated signatures within classes of cells, yields a new slate of gene candidates for further study. Cell surface markers, in particular, may lead a new class of targetable molecules to modulate or mitigate cell type changes observed through sc/snRNA-seq and other modalities in brain tissue. Thus, the refinement of molecular profiling methods in terms of modality and spatial context is an important frontier in cell type targeting-based approaches for disease management.

## Conclusions

Although relatively new, the profiling of human brain tissue in AD using sc/snRNA-seq has already revealed multiple cell type-specific alterations associated with disease phenotypes. In addition to characterizing vulnerable and resistant neuronal subclasses—which is challenging to do in bulk studies—the selective changes in cerebrovascular and glial signatures show a host of systemic effects associated with AD. Although these studies cannot in themselves establish causality without the use of alternative techniques such as Mendelian randomization [28, 78] or validation using gene perturbations in model systems [71, 72], they still highlight key cell type-specific signatures that may lead to the identification of surface molecules and targets for therapeutics.

With new profiling methods and much larger sample sizes on the horizon, this picture of cell type-specific changes will be continuously refined. The introduction of new techniques to profile cells in intact tissues, combined with the multi-modal dissociated single-cell profiling approaches described here, will further identify putative associations between cell signatures and pathology, as well as potential cell-cell interactions (Fig. 4). These new frontiers in tissue profiling and analysis will help narrow down potential target genes and pathways for clinical investigation; whereas single-cell approaches have narrowed down bulk profiling targets to specific cell types, spatial approaches and larger sample sizes will further refine these targets with increased statistical confidence and context with respect to pathological features. By expanding clinical studies to include cell type perturbations, in combination with pathology-clearing measures, these types of studies are likely to prioritize additional therapeutic candidates for pre-clinical studies, leading ultimately to more varied therapies to stratify, monitor, and combat the effects of AD pathology on cognitive function.

## Data Availability

Not applicable.
